# Elastomeric microparticles for acoustic mediated bioseparations

**DOI:** 10.1186/1477-3155-11-22

**Published:** 2013-06-28

**Authors:** Leah M Johnson, Lu Gao, C Wyatt Shields IV, Margret Smith, Kirill Efimenko, Kevin Cushing, Jan Genzer, Gabriel P López

**Affiliations:** 1 Department of Biomedical Engineering, Duke University, 101 Science Drive, 3361 CIEMAS, Durham, NC, 27708, USA; 2 Department of Mechanical Engineering and Materials Science, Duke University, Box 90300 Hudson Hall, Durham, NC, 27708, USA; 3 NSF Research Triangle Materials Research Science and Engineering Center, Duke University, Box 90271, Durham, NC, 27708, USA; 4 Department of Chemical and Biomolecular Engineering, North Carolina State University, Engineering Building 1, 911 Partners Way, Raleigh, NC, 27695, USA; 5 Center for Biomedical Engineering, University of New Mexico, 210 University Blvd NE, Albuquerque, NM, 87131, USA; 6 National Flow Cytometry Resource, Los Alamos National Laboratory, Los Alamos, NM, 87545, USA

**Keywords:** Cell separation, Continuous cell sorting, Acoustofluidics, Particle synthesis, Ultrasound standing wave

## Abstract

**Background:**

Acoustophoresis has been utilized successfully in applications including cell trapping, focusing, and purification. One current limitation of acoustophoresis for cell sorting is the reliance on the inherent physical properties of cells (e.g., compressibility, density) instead of selecting cells based upon biologically relevant surface-presenting antigens. Introducing an acoustophoretic cell sorting approach that allows biochemical specificity may overcome this limitation, thus advancing the value of acoustophoresis approaches for both the basic research and clinical fields.

**Results:**

The results presented herein demonstrate the ability for negative acoustic contrast particles (NACPs) to specifically capture and transport positive acoustic contrast particles (PACPs) to the antinode of an ultrasound standing wave. Emulsification and post curing of pre-polymers, either polydimethylsiloxane (PDMS) or polyvinylmethylsiloxane (PVMS), within aqueous surfactant solution results in the formation of stable NACPs that focus onto pressure antinodes. We used either photochemical reactions with biotin-tetrafluorophenyl azide (biotin-TFPA) or end-functionalization of Pluronic F108 surfactant to biofunctionalize NACPs. These biotinylated NACPs bind specifically to streptavidin polystyrene microparticles (as cell surrogates) and transport them to the pressure antinode within an acoustofluidic chip.

**Conclusion:**

To the best of our knowledge, this is the first demonstration of using NACPs as carriers for transport of PACPs in an ultrasound standing wave. By using different silicones (i.e., PDMS, PVMS) and curing chemistries, we demonstrate versatility of silicone materials for NACPs and advance the understanding of useful approaches for preparing NACPs. This bioseparation scheme holds potential for applications requiring rapid, continuous separations such as sorting and analysis of cells and biomolecules.

## Background

Microparticles suspended in an ultrasound standing wave field may respond to the primary acoustic radiation force by transporting to specific locations along the wave (i.e., pressure node or pressure antinode) [1-4]. The primary radiation force (*F*) exerted on a particle depends on several factors including the acoustic pressure amplitude (*P*_0_), particle volume (*V*_p_), wavelength (*λ*), and the acoustic contrast factor (ϕ) (Equation 1, where *k* is the wavenumber and *x* is the distance from a vertical wall of the microfluidic channel). Importantly, the sign of the acoustic contrast factor, which depends on both the density (*ρ*) and the compressibility (*β*) of the particle relative to the surrounding solution, dictates the relocation (Equation 2). For example, particles with higher compressibility (*β*_p_) than the surrounding media (*β*_w_) will move to the pressure antinode, whereas particles with a lower compressibility than the surrounding media will move to the pressure node. In general, particles with a positive ϕ (i.e., PACPs), such as polystyrene beads or cells, transport to acoustic pressure nodes within aqueous media, whereas particles with a negative ϕ (i.e., NACPs) transport to the acoustic pressure antinodes within aqueous media.

(1) $$F=\left(\frac{\pi P_{0}^{2}V_{p}\beta_{w}}{2\lambda }\right)∙\phi \left(\beta ,\rho \right)∙\sin\left(2\mathrm{kx}\right)$$

(2) $$\phi \left(\beta ,\rho \right)=\frac{5\rho_{p}-2\rho_{w}}{2\rho_{p}+\rho_{w}}-\frac{\beta_{p}}{\beta_{w}}$$

The capacity to relocate PACPs to pressure nodes has been used in various approaches for focusing and separation of mammalian cells [5-11]. For example, the recently commercialized Attune® flow cytometer (Life Technologies) substitutes traditional hydrodynamic focusing with ultrasonic standing wave fields to focus cells into a single flowing stream prior to laser interrogation [5]. To increase the high-throughput capacity of flow cytometry, Piyasena *et al*. recently developed multi-node acoustic focusing and demonstrated up to 37 parallel flow streams [6]. Peterson *et al*. exploited the inherent contrast factor of constituents from whole blood to separate and sort positive contrast erythrocytes from negative contrast lipids within an acoustofluidic device [7,8]. Strategies for separating two particle populations with contrast factors of the same sign can exploit differences in the magnitude of the acoustic force [9,10]. In certain cases, the contrast factor can be adjusted by changing the density of the solution, as shown in a report separating polystyrene and PMMA microparticles by increasing the salt concentration of the media [11].

**Figure 1:**
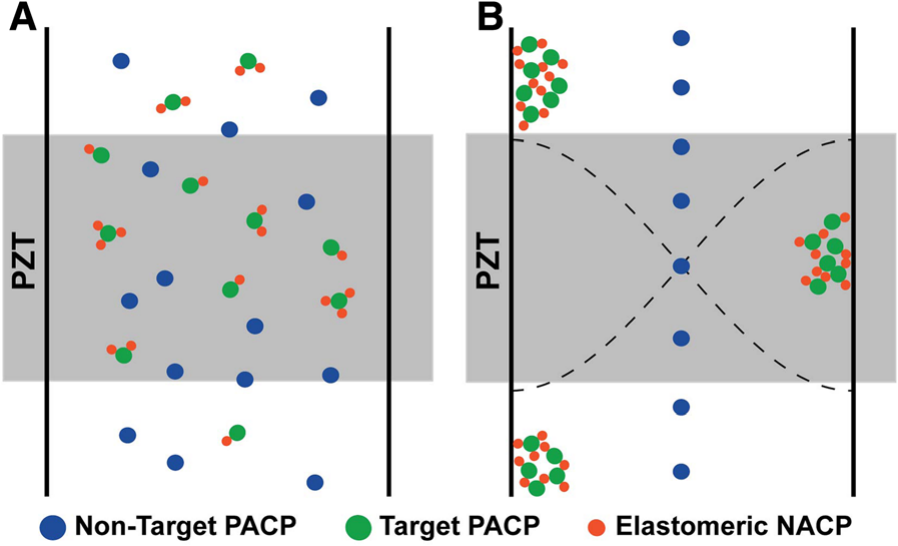
**Acoustic mediated bioseparation using NACPs.** Schematic illustrating the use of NACPs as carriers for directed transport of PACPs (e.g., cells). (**A**) In the absence of the acoustic standing wave (PZT off), all particles distribute randomly within the acoustofluidic channel. (**B**) In the presence of the acoustic standing wave (PZT on), microparticles transport either to the pressure node (solitary non-targeted PACPs, blue) or to the acoustic anti-node (NACPs, red). Here, the acoustofluidic channel operates at a half wavelength resonant mode perpendicular to flow resulting in an antinode at both channel walls and a single node in the middle of the channel. By designing NACPs with biological affinity for targeted PACPs (green), NACP-PACP complexes form and collectively transport to the pressure antinode. Sorted PACPs may be collected downstream using a trifurcation configuration. Schematic is not to scale and represents conditions without flow or low flow rates.

 We seek to augment acoustophoretic particle sorting capabilities by introducing newly designed negative acoustic contrast particles (NACPs) with the capacity for specific biomolecular recognition and relocation of PACPs to antinodes of ultrasound standing waves. Since NACPs move in the direction opposite to the majority of mammalian cells, we hypothesized that biofunctional NACPs can capture and specifically transport targeted cells (or other PACPs) to the pressure antinodes, provided the total acoustic force of the NACPs is greater than the total acoustic force of the PACPs. Figure 1 illustrates the principle. Central to this bioseparation scheme is the specific association between the engineered NACPs and targeted PACPs to create a stable complex capable of in-tandem transport to the pressure antinode. This requires precise design of biofunctional NACPs that exhibit stability and specificity for targeted PACPs. Recently, Cushing *et al*. reported the first use of NACPs for biomolecule quantification assays by using protein adsorption to modify the surface of PDMS particles [12]. While convenient, such adsorption techniques often generate heterogeneous surfaces resulting from random orientation and denaturation of proteins on the surface [13]. These considerations become more important in cell sorting applications that require high concentrations of active, surface-presenting bioaffinity groups for capturing rare cells and cells with a low quantity of targeted surface antigens.

Herein, we report on the preparation of NACPs and demonstrate the utility of these microparticles in a new acoustophoretic separation scheme. Specifically, NACPs are prepared using two different silicone elastomers and biotinylated using two different chemical modification approaches. The newly designed NACPs are evaluated as carriers for the transport of streptavidin PACPs to pressure antinodes within acoustofluidic devices. Our results reveal the potential of this approach for cell sorting applications.

## Results and discussion

### Silicone microparticles as biofunctional NACPs

**Figure 2:**
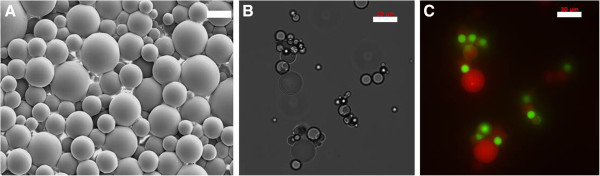
**Silicone NACPs for acoustic mediated bioseparations.** (**A**) SEM image of NACPs comprising PDMS. Brightfield image (**B**) and the accompanying fluorescence image (**C**) of biotinylated PDMS particles (red) binding streptavidin polystyrene microparticles (green, 6 μm diameter). PDMS particles are encapsulated with rhodamine B and surface-functionalized with biotin-Pluronic F108. Scale bars represent 20 μm.

Silicone elastomers offer properties suitable for NACPs such as compressibility at mild temperature (e.g., Young’s modulus ~1MPa for typical PDMS formulations) [14]. Here, all NACPs were prepared by emulsifying silicone pre-polymers in aqueous surfactant solutions and subsequently curing to produce solid microparticles (Figure 2A). Because homogenization produces polydisperse particles, filtration or centrifugation was employed to narrow the breadth of particle size distributions. In one example, filtration of NACPs with a 12 μm polycarbonate filter resulted in an average particle diameter of 6 ±3 μm (Additional file 1). Although a variety of surfactants enabled formation of silicone-in-water emulsions, the importance of surfactant type became evident when attempting to re-suspend cured NACPs in surfactant-free buffer, which often resulted in irreversible particle aggregation. Here, we found that the block copolymer surfactant, Pluronic® F108, stabilizes silicone microparticles likely due to the strong association of the hydrophobic polypropylene oxide block with silicone [15]. We further exploited this stable association by end-functionalizing Pluronic® F108 with biotin (Figure 2B,C). Biotin-Pluronic F108 enables use of the streptavidin protein as a linker between NACPs and any biotinylated analyte (e.g., cells labelled with biotinylated antibodies).

**Figure 3:**
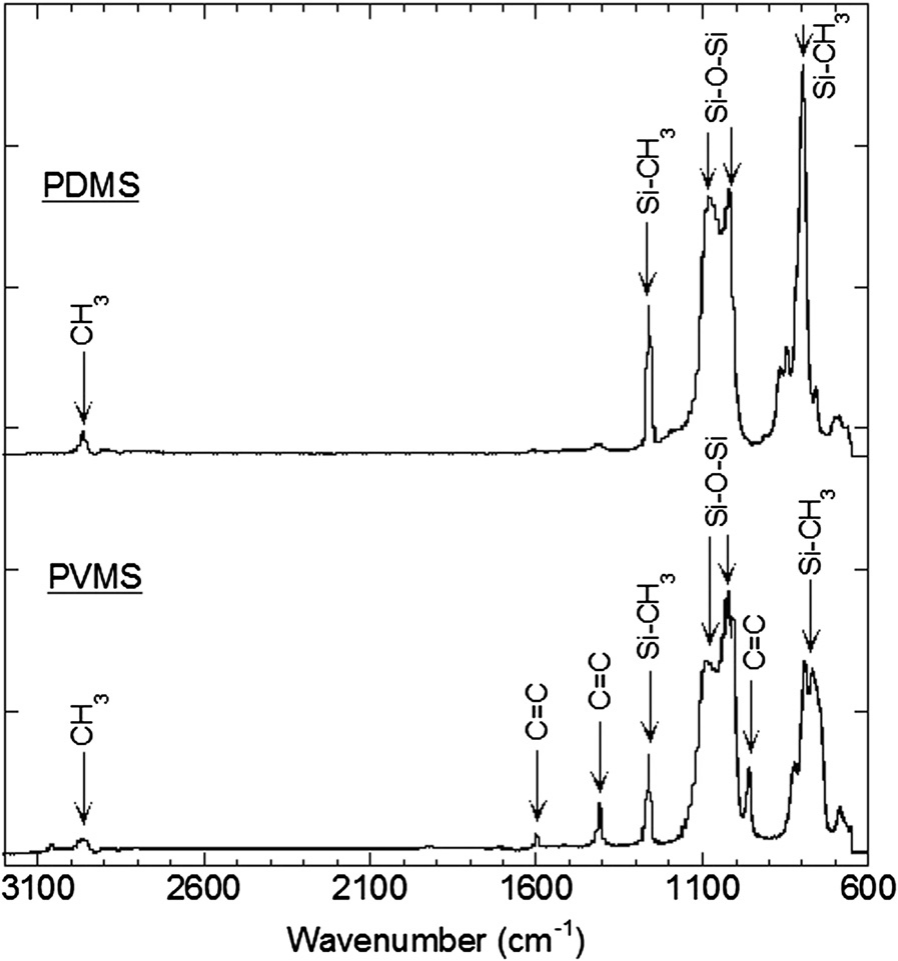
**ATR-FTIR spectra of PDMS and PVMS.** PDMS and PVMS exhibit IR peaks at 789–796 cm^-1^ (−CH_3_ rocking and Si-C stretching in Si-CH_3_), 1020–1074 cm^-1^ (Si-O-Si stretching), 1260–1259 cm^-1^ (CH_3_ deformation in Si-CH_3_), and 2950–2960 cm^-1^ (asymmetric CH_3_ stretching in Si-CH_3_). The spectra for PVMS shows IR peaks characteristic for C=C at 958 cm^-1^, 1408 cm^-1^, and 1597 cm^-1^.

**Figure 4:**
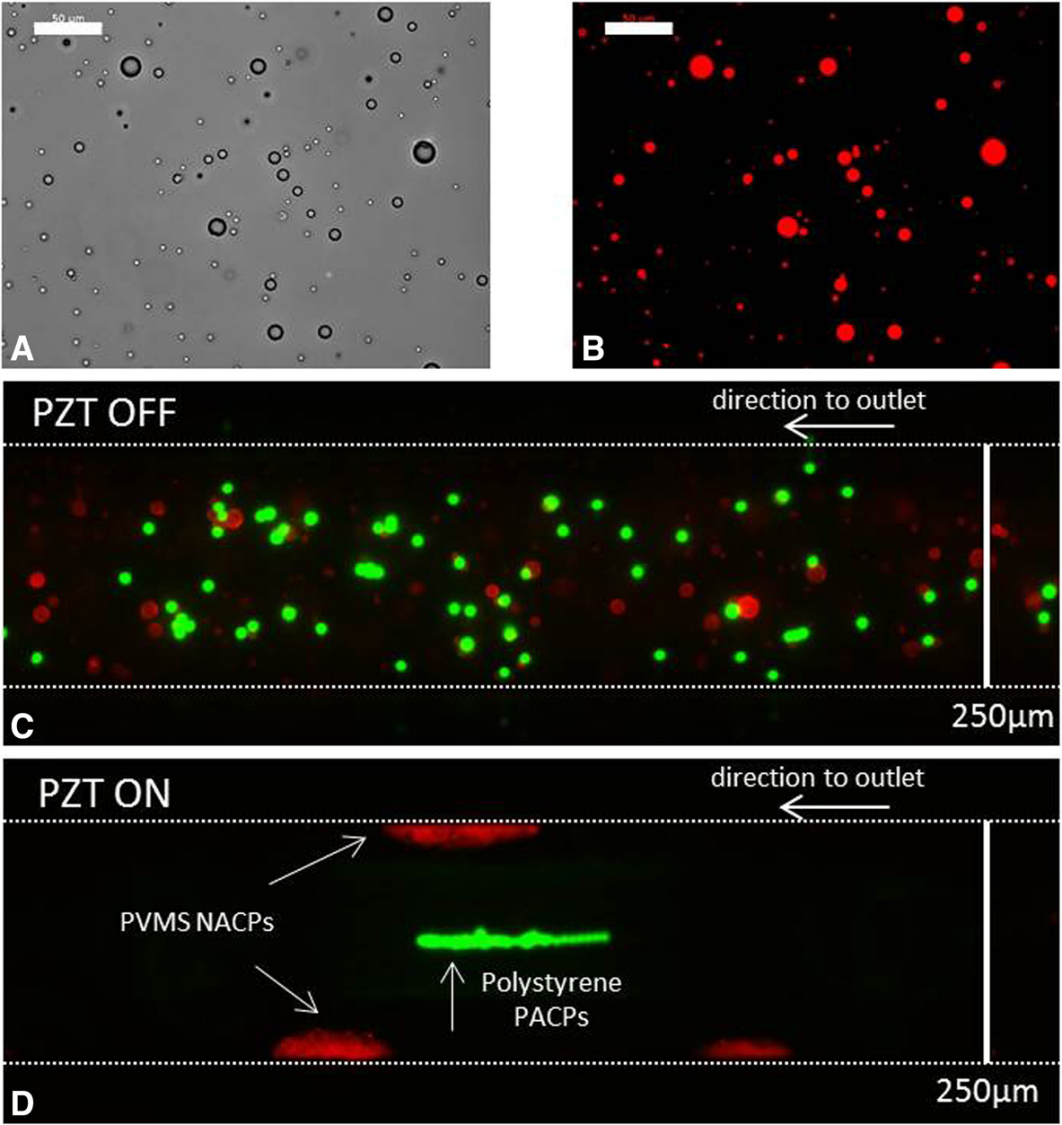
**Acoustic response of silicone NACPs.** Brightfield image (**A**) and corresponding fluorescence image (**B**) of PVMS microparticles functionalized with biotin-TFPA and subsequently labelled with streptavidin Alexa Fluor® 488. The fluorescent image was acquired during a 250 ms exposure. The scale bars represent 50 μm. (**C**, **D**) Fluorescence images show a mixture of PVMS microparticles (red, functionalized with biotin-TFPA and streptavidin Alexa Fluor® 546) and polystyrene microparticles (green, non-biotinylated, Spherotech, 10–13 μm diameter) within a channel of an acoustofluidic device with (**C**) and without (**D**) activation of the PZT. Mixture contained a 1:7 ratio of polystyrene:PVMS microparticles. Images acquired in the absence of flow. Dashed lines are included to demarcate the channel boundaries.

We also sought to evaluate the feasibility of direct modification of NACPs. Typically, surface modification of PDMS is accomplished by employing modification methods such as ultraviolet (UV)/ozone irradiation [16], UV graft polymerization [13], oxygen plasma treatment [17], and adsorption [18]. These modification approaches are usually performed on macroscopic silicone surfaces not held to the unique stringencies required to functionalize NACPS. For NACPs, conditions must be avoided that cause significant change in modulus or irreversible microparticle aggregation. For instance, modification of PDMS surfaces via plasma treatment results in the formation of brittle silica layers [19] which could affect the negative acoustic contrast property. Here, to evaluate direct, covalent modification of particles, we used PVMS which contains vinyl groups and can be functionalized chemically without forming a silica-like crust [16]. To first evaluate and compare chemical groups in both PDMS and PVMS, bulk samples were prepared and characterized using ATR-FTIR (Figure 3). PVMS material displays characteristic vinyl peaks at 958 cm^-1^ (C=C twist, =CH_2_ wagging), 1,408 cm^-1^ (=CH_2_ scissors), and 1,597 cm^-1^ (C=C stretch). While vinyl groups are versatile for various chemical reactions (e.g., thiolene or methathesis coupling), our studies revealed that relatively simple photochemical reaction with biotin-TFPA results in biofunctionalization of PVMS particles (Figure 4A,B). Photoreacting biotin-TFPA with PVMS microparticles and subsequently adding fluorescent streptavidin resulted in significant differences in fluorescent signal between positive and negative samples (Additional file 2). For example, signal to background values (S/B) of fluorescent images of PVMS microparticles functionalized with biotin-TFPA and fluorescent streptavidin was 22 ± 2, whereas the negative control reaction without light irradiation was 9.0 ± 0.3, suggesting a biotinylation reaction of NACPs occurred. Notably, these studies cannot discern the exact location of biotinylation (e.g., vinyl groups or associated surfactant), as TFPA may react with C-H, N-H, or C=C groups [20]. Initial attempts at using biotin-TFPA to functionalize PDMS microparticles resulted in similar trends with a S/B values of 22 ± 3, supporting the non-specificity of biotin-TFPA. Overall, these studies demonstrate the utility of using biotin-TFPA for bio-functionalization of silicone microparticles.

**Figure 5:**
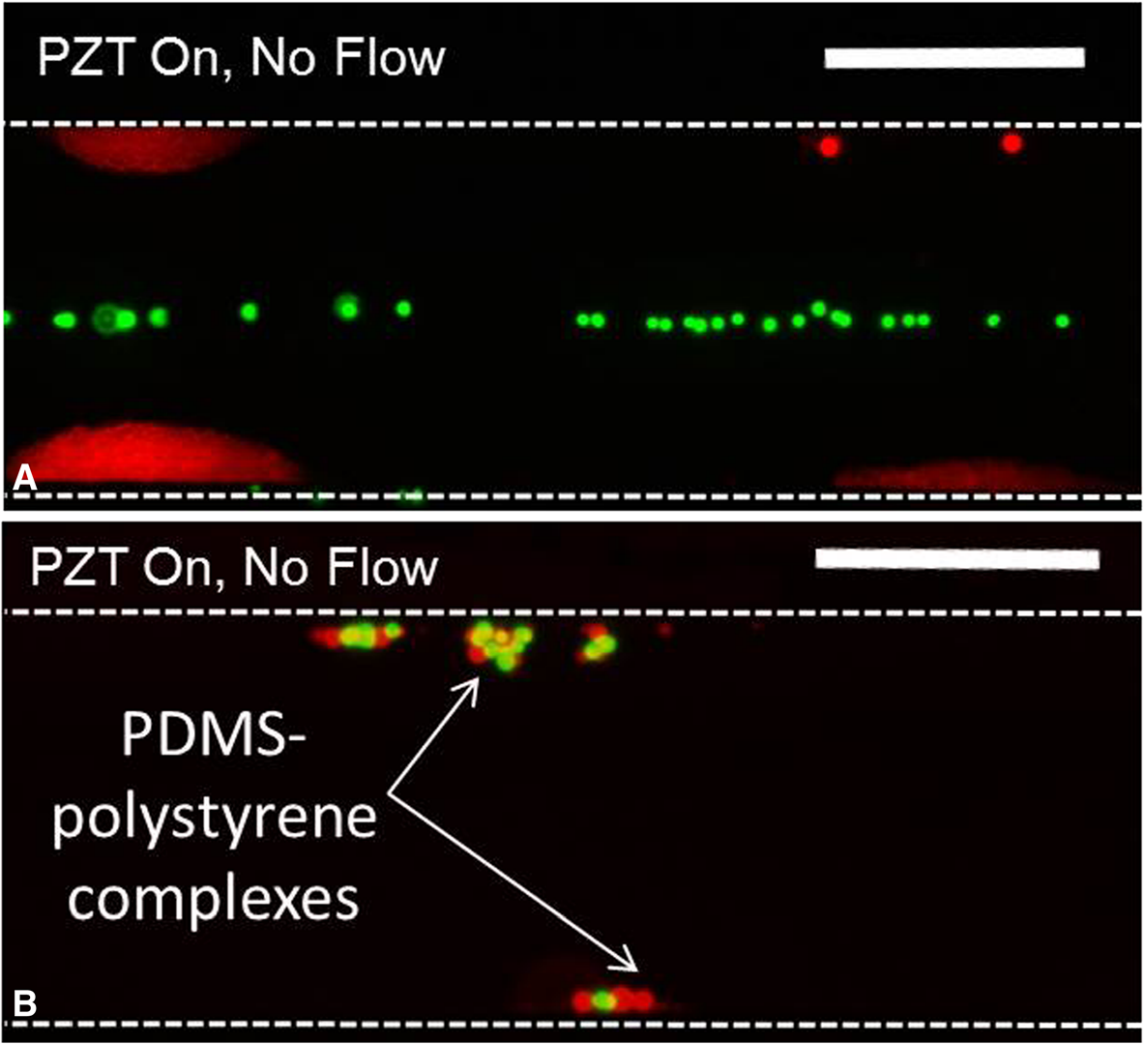
**Using NACPs to transport PACPs to the pressure antinode.** Fluorescence images demonstrate the ability to use NACPs to transport PACPs to the pressure antinode within an acoustofluidic device. (**A**) As a negative control, PDMS microparticles (non-biotinylated, encapsulated with Nile Red fluorophore) were mixed with streptavidin polystyrene microparticles (green, 6 μm diameter). The lack of binding between the non-biotinylated PDMS and streptavidin polystyrene particles results in their transport to the antinode and node, respectively. (**B**) The high affinity between PDMS microparticles (biotinylated, encapsulated with rhodamine B fluorophore) and streptavidin polystyrene microparticles (green, 6 μm diameter) generate particle complexes that transport collectively to the pressure antinode within an ultrasound standing wave. Images acquired in the absence of flow with a 1:10 ratio of polystyrene:PDMS. Dashed lines are included to demarcate the channel boundaries. Scale bars represent 200 μm.

Next, we sought to evaluate the acoustic responsiveness of these silicone microparticles. Our results show that microparticles prepared from either PVMS or PDMS function as NACPs within aqueous media (Figures 4 and 5). For example, a mixture of biotinylated PVMS NACPs and non-biotinylated polystyrene microparticles randomly distribute within an acoustofluidic channel in the absence of a standing wave field (Figure 4C). Upon application of an operating frequency of 2.98 MHz to generate an ultrasound standing wave within the microchannel (wavelength = 2 × channel width), polystyrene and PVMS microparticles separate (Figure 4D). Here, particle separation occurred in less than one second as determined under this experimental setup. Determining the precise rate of particle separation would require further measurements with a high speed camera to track trajectories of individual particles. Incompressible positive acoustic contrast polystyrene particles transport to the center of channel, corresponding to the pressure node, whereas compressible PVMS NACPs transport to the channel sidewalls, corresponding to the pressure antinodes. The capacity for both PDMS and PVMS to function as NACPs (Figures 4 and 5) illustrates the versatility of using silicone elastomers with different chemical compositions. Although only two silicone materials were tested here, we envision schemes to enhance the repertoire of available functional groups by employing a range of functional silicones that could be used for bioconjugation reactions. It is important to note is that the “PDMS” microparticles studied here (i.e., Sylgard 184, which is a blend of PDMS, silica, and resin fillers) exhibited negative acoustic contrast despite containing silica fillers.

### NACPs as carriers for acoustic-mediated separations

The separation of silicone NACPs from polystyrene microparticles demonstrated in Figure 4 encouraged further investigations aimed at evaluating potential for the use of NACPs in cell separations. We hypothesized that NACP-PACP complexes within aqueous media will transport to pressure antinodes, provided the total radiation force from NACPs in the complex is greater than the total radiation force from PACPs in the complex. To this end, we employed polystyrene microparticles as surrogates for mammalian cells and investigated separation characteristics using NACPs prepared from PDMS. The brightfield image (Figure 2B) and accompanying fluorescent image (Figure 2C) show association between streptavidin coated polystyrene and PDMS microparticles functionalized with biotin-Pluronic F108. Notably, within the acoustofluidic device, the NACP-polystyrene microparticle complexes transport in unison to the pressure antinode (Figure 5B). This supports the notion that NACPs may serve as vehicles for specific transport of positive acoustic contrast particles. Conversely, non-biotinylated PDMS microparticles did not bind streptavidin polystyrene particles. This is shown in the negative control (Figure 5A) where non-biotinylated PDMS particles (red) transport to the pressure antinode and polystyrene microparticles (green) align at the pressure node. Figure 5 suggests the feasibility of a new bioseparation technique where transport of targeted PACPs (e.g., cells) will rely on specific, well-defined interactions with the NACPs. Figure 5 shows all PACP-NACP complexes transported to the antinode at the acoustofluidic wall (e.g., ~14 NACPs and ~12 PACPs in four separate complexes). However, additional studies are required to further understand the effects of parameters, such as particle ratios, flow rates, and applied voltages on efficiency of separation.

As expected, in the absence of fluid flow, NACPs accumulate at the pressure antinodes along the acoustofluidic channel walls during activation of the PZT (Figures 4 and 5). Secondary acoustic forces contribute to the aggregation of NACPs, as previously described for lipids in milk emulsions and whole blood [7,8,21]. This NAPC aggregation may be reduced by introducing flow to the channel. As recently demonstrated, laminar flow within the channel enables NACPs to maintain their position at the pressure antinode while simultaneously moving along laminar streamlines to the downstream trifurcation [12]. This capacity to couple relocation with downstream sample collection facilitates continuous sorting applications.

To the best of our knowledge, this is the first report documenting the use of NACPs as carriers for active transport of PACPs in acoustofluidic systems. Although polystyrene microparticles were used as cell surrogates in this preliminary investigation to demonstrate separation, this approach should be suitable for cell sorting based on binding of NACPs to specific cell surface antigens. Because the positive acoustic contrast factor value of cells is less than polystyrene beads [22], we anticipate that cell-NACP complexes should readily transport to pressure antinodes. Thus, this method holds potential as a complement to current cell sorting techniques (e.g., fluorescence-activated or magnetic-activated cell sorting). In contrast to these conventional methods, the present technique offers the possibility of enhanced selectivity and separation efficiency since ultrasound wave fields exert forces on *both* NACPs and PACPs in opposing directions. Given this promise, it is necessary to further examine several aspects of using NACPs in cellular separations. For example, the role of bioaffinity bond strength between particles that are being subjected to force in opposite directions may need to be studied in detail. Likewise, the features that enable the primary radiation force of NACPs to dominate that of PACPs requires further investigation. The transport of PACPs to the pressure antinodes will only occur when a complex of PACP bound to NACPs exhibits an overall negative acoustic contrast factor, which can be adjusted through the volume, density, and bulk modulus of the NACPs. In the current study, these properties have converged to favor the relocation of PACP-NACP complexes to the antinode. We anticipate that future experimental and computational investigations will reveal the optimal parameters that support efficient cell separation.

## Conclusions

This report communicates a new approach for bioseparation that employs polysiloxane-based microparticles with a negative acoustic contrast property. Emulsifying and post-curing pre-polymers within aqueous surfactant results in stable microparticles that transport to the pressure antinode of an ultrasonic standing wave field in aqueous media. By using polysiloxanes with different chemical compositions and curing chemistries (i.e., PDMS, PVMS), we demonstrate versatility and general utility of silicone materials as negative acoustic contrast agents. Both photochemical and physical adsorption approaches are used to biofunctionalize NACPs, ultimately enabling the specific capture and transport of PACPs to an acoustic pressure antinode. These results encourage further pursuits aimed at using NACPs for cell separation, owing to potential advantages of this system such as high sensitivity, selectivity, portability and low cost.

## Methods

### Preparation & functionalization of NACPs

*Preparing PVMS particles*: A mixture of 1.0 g of hydroxyl-terminated PVMS [14], 0.07 g vinylmethoxysiloxane homopolymer (Gelest), and between 0.02 g and 0.03g tin octoate catalyst (Gelest) was thoroughly stirred and combined with a solution of 0.5 or 0.7 wt% Pluronic® F108 (Aldrich) in ultrapure water (Mill-Q, 18MΩ resistivity). The mixture was briefly vortexed, homogenized using a PT 1200E homogenizer (Polytron) with a 3mm rotor for 5 min at 18,750 rpm, and stirred for at least 2 hr at ~50°C. The polydisperse emulsion was permitted to cure via alkoxy condensation of silanol-terminated PVMS with vinylmethoxysiloxane. Particles were left at ambient conditions for approximately one week, then filtered through a 12 μm polycarbonate membrane (Whatman, Cyclopore), and stored at ambient conditions until use. *Preparing PDMS particles*: A mixture comprising a 1:10 weight ratio of curing agent: base of Sylgard® 184 (Dow Chemical) was thoroughly mixed and 1 gram of the mixture was subsequently combined with 1 wt% of Pluronic F108. The mixture was homogenized as previously described. The emulsion was incubated at 45°C, stirring for at least 1.5 hrs and subsequently left at ambient conditions for at least 12 hrs to permit curing. *Functionalization*: For reactions with biotin-TFPA (Quanta Biodesign), ~5 × 10^7^ PVMS microparticles were washed with 1× PBS by centrifuging and resupending the pellet in a final volume of 2 mL of 1× PBS. The microparticles were transferred to a cylindrical glass vial (2.5 cm diameter) and 3 mg biotin-TFPA in 100 μL of dimethylacetamide was added. Light irradiation occurred using an Omnicure S1000 equipped with a high pressure mercury lamp and an internal 320–500 nm filter. The associated light guide was placed ~5 mm above the stirring solution for 30 min at a light intensity of ~100 mW/cm^2^ at a wavelength of 365 nm, (as measured by Powermax USB sensor, Coherent). The resultant yellow solution was stored at 4°C until use. Biotinylation of Pluronic F108 surfactant followed a similarly reported protocol [20]. Briefly, hydroxyl end groups on F108 were modified to succinimidyl carbonate using N,N’-disuccinimidyl carbonate (Aldrich) and 4-(dimethylamino)pyridine (Aldrich) and subsequently reacted with biotin-hydrazide (Aldrich). Once biotinylated, Pluronic F108 was used to prepare silicone emulsions as previously described. Subsequent addition of streptavidin (AlexaFluor® 488 or AlexaFluor® 546) to NACPs occurred by washing particles at least three times by centrifuging and resuspending the pellet in 1× PBS, and incubating with either 1 μM or 1.7 μM of streptavidin for 30 min at room temperature.

### Characterization of negative acoustic contrast materials and microparticles

Attenuated total reflection-Fourier transform infrared (ATR-FTIR) spectra were acquired using a Thermo Electron Nicolet 8700 spectrometer (Ge crystal, 32 scans, 4 cm^2^ resolution). Scanning electron microscopy (SEM) images were obtained using model FEI XL 30 SEM under ultra-high resolution mode after sputter coating the samples with approximately 6 nm of gold. Optical microscopy images were obtained using an upright Zeiss Axio Imager A2 microscope with appropriate filter set (ex 470/40, em 525/50 or ex 545/25, em 605/70 or ex 365, em 445/50).

### Bioseparation studies

Binding between streptavidin polystyrene microparticles (Polysciences, YG microspheres, 6 μm) and PDMS NACPs (encapsulated with rhodamine B, functionalized with biotin-F108) occurred by combining ~10^6^ polystyrene particles and ~10^7^ PDMS particles and incubating for 30 minutes at room temperature with end-over-end rotation. Before combining with the polystyrene microparticles, ~10^7^ PDMS NACPs were washed three times with 1× PBS. Polystyrene particles were added directly from the manufacturer’s stock without washing. Bioseparation events within the channel were monitored through the glass lid of the acoustofluidic device using fluorescent microscopy.

### Fabrication of acoustofluidic device

The acoustofluidic device (Additional file 3) was prepared using standard photolithography, deep reactive-ion etching, anodic bonding and plasma bonding. The device contained a downstream collection module and an acoustic (piezoelectric) actuation element (i.e., lead zirconate titanate, PZT, 841 WFB, d_33_ = 0.3 nm/V, APC International). The channel width was designed to operate at a half wavelength resonant mode (e.g., 252 μm and frequency of 2.94 MHz or 272 μm and frequency of 2.72MHz) resulting in an antinode at both channel walls and a single node in the channel center line. For the experiments, an electric signal with peak-to-peak voltage of 31 V was applied to the PZT. Prior to running experiments, the acoustofluidic channels were treated with a solution of Pluronic F108.

## Abbreviations

PACPs: Positive acoustic contrast particles; NACPs: Negative acoustic contrast particles; PBS: Phosphate buffered saline; PDMS: Polydimethylsiloxane; PVMS: Polyvinylmethylsiloxane; TFPA: Tetrafluorophenyl azide.

## Competing interests

The authors declare no competing interests.

## Authors’ contributions

LMJ, LG, CWS, and MS performed the experiments including the microparticle preparation and characterization, acoustofluidic chip fabrication, and bioseparation experiments. KE prepared and characterized PVMS material and contributed to PVMS particle formulation. KC helped design preparation procedures for PDMS particles. All authors offered scientific input during these studies and assisted in preparation of the manuscript. All authors approved the manuscript.

## Supplementary Material

Additional file 1 **Size distribution of NACPs after filtration.** This graph shows the size distribution (6 ± 3 μm) of PVMS NACPs (prepared with 0.3 wt% cetyltrimethylammonium bromide surfactant) after filtration with a 12 μm polycarbonate filter. The particle diameters were determined using optical microscopy with a 40× objective. Any microparticles <1 μm would not be resolved with this technique. Click here for file

Additional file 2 **Analysis of PVMS microparticles after functionalization with biotin-TFPA and fluorescent streptavidin.** Histogram showing the signal to background (S/B) fluorescent values of: (A) PVMS microparticles (~5 × 10^7^ particles/mL) combined with biotin-TFPA and irradiated with a 320–500 nm light source (~10 mW/cm^2^) for 30 minutes. The particles were subsequently labeled with streptavidin AlexaFluor® 488 and washed with 1× PBS. The negative control reactions were performed identically except (B) without light irradiation or (C) without biotin-TFPA. All fluorescent values were taken from images acquired using a 40× objective and 25 ms exposure. Three separate fluorescent images from the same sample were taken and used to calculate standard deviations. Click here for file

Additional file 3 **Acoustofluidic device.** Digital camera images showing (A) the glass top and (B) the silicon underside of an exemplary acoustofluidic device. To collect downstream sorted particles, a trifurcation arrangement was designed with two side outlets and a single middle outlet, where negative and positive acoustic contrast particles would exit, respectively. The PZT is attached to the silicon underside. Click here for file
