# Supplementary figures and images for: Elastomeric microparticles for acoustic mediated bioseparations
Source: J Nanobiotechnology. 2013 Jun 28;11:22. doi: 10.1186/1477-3155-11-22 (PMC3706277; doi:10.1186/1477-3155-11-22)

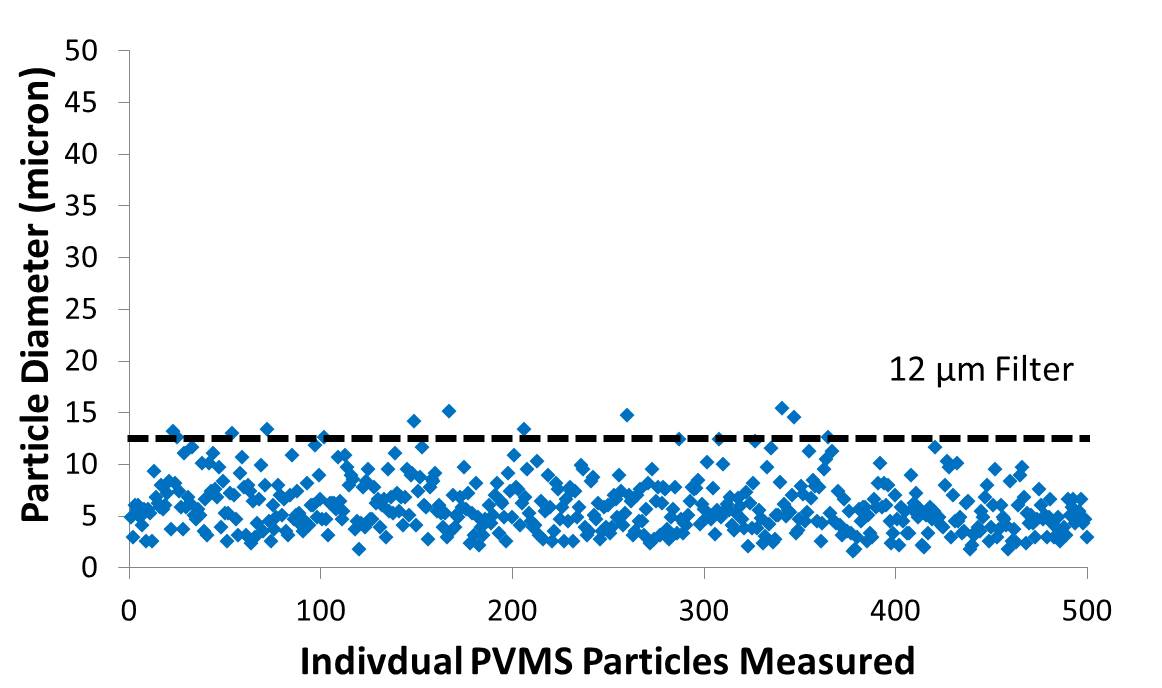

Supplement: Additional file 1 — Size distribution of NACPs after filtration. This graph shows the size distribution (6 ± 3 μm) of PVMS NACPs (prepared with 0.3 wt% cetyltrimethylammonium bromide surfactant) after filtration with a 12 μm polycarbonate filter. The particle diameters were determined using optical microscopy with a 40× objective. Any microparticles <1 μm would not be resolved with this technique. [file 1477-3155-11-22-S1.jpeg]

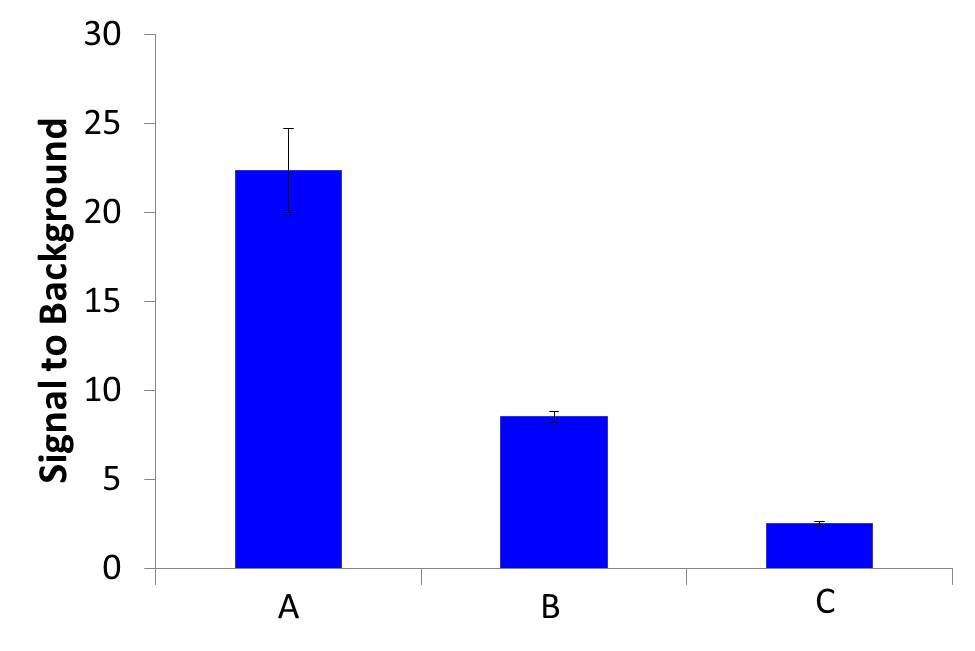

Supplement: Additional file 2 — Analysis of PVMS microparticles after functionalization with biotin-TFPA and fluorescent streptavidin. Histogram showing the signal to background (S/B) fluorescent values of: (A) PVMS microparticles (~5 × 10^7^ particles/mL) combined with biotin-TFPA and irradiated with a 320–500 nm light source (~10 mW/cm2) for 30 minutes. The particles were subsequently labeled with streptavidin AlexaFluor® 488 and washed with 1× PBS. The negative control reactions were performed identically except (B) without light irradiation or (C) without biotin-TFPA. All fluorescent values were taken from images acquired using a 40× objective and 25 ms exposure. Three separate fluorescent images from the same sample were taken and used to calculate standard deviations. [file 1477-3155-11-22-S2.jpg]

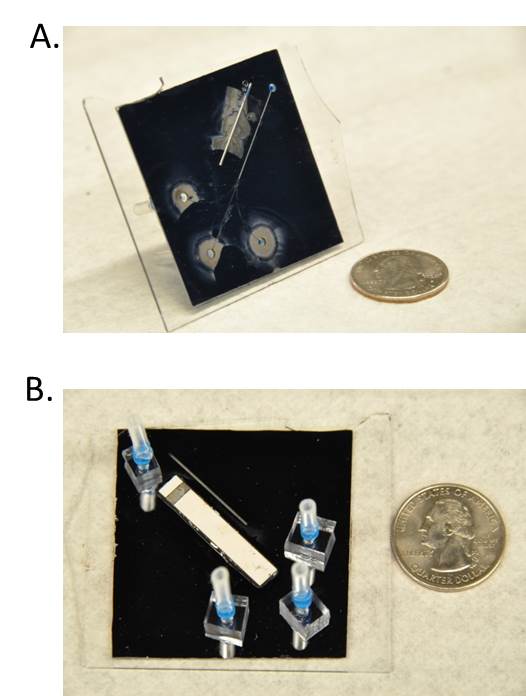

Supplement: Additional file 3 — Acoustofluidic device. Digital camera images showing (A) the glass top and (B) the silicon underside of an exemplary acoustofluidic device. To collect downstream sorted particles, a trifurcation arrangement was designed with two side outlets and a single middle outlet, where negative and positive acoustic contrast particles would exit, respectively. The PZT is attached to the silicon underside. [file 1477-3155-11-22-S3.jpeg]
